# Role of Conserved Residues and F322 in the Extracellular Vestibule of the Rat P2X7 Receptor in Its Expression, Function and Dye Uptake Ability

**DOI:** 10.3390/ijms21228446

**Published:** 2020-11-10

**Authors:** Marian Rupert, Anirban Bhattacharya, Vendula Tvrdonova Stillerova, Marie Jindrichova, Audrey Mokdad, Eric Boué-Grabot, Hana Zemkova

**Affiliations:** 1 Institute of Physiology, Czech Academy of Sciences, 14220 Prague, Czech Republic; Marian.Rupert@fgu.cas.cz (M.R.); anirban.bee@gmail.com (A.B.); v.stillerova@gmail.com (V.T.S.); M.Jindrichova@seznam.cz (M.J.); audrey.mokdad@gmail.com (A.M.); 2 1st Faculty of Medicine, Charles University, 12108 Prague, Czech Republic; 3 Institute des Maladies Neurodégénératives, University de Bordeaux, UMR 5293, F-33000 Bordeaux, France; eric.boue-grabot@u-bordeaux2.fr; 4 Centre National de la Recherche Scientifique, UMR 5293, F-33000 Bordeaux, France

**Keywords:** P2X7 receptor, extracellular vestibule, mutagenesis, gating, deactivation, dye uptake, HEK293T cells

## Abstract

Activation of the P2X7 receptor results in the opening of a large pore that plays a role in immune responses, apoptosis, and many other physiological and pathological processes. Here, we investigated the role of conserved and unique residues in the extracellular vestibule connecting the agonist-binding domain with the transmembrane domain of rat P2X7 receptor. We found that all residues that are conserved among the P2X receptor subtypes respond to alanine mutagenesis with an inhibition (Y51, Q52, and G323) or a significant decrease (K49, G326, K327, and F328) of 2′,3′-O-(benzoyl-4-benzoyl)-ATP (BzATP)-induced current and permeability to ethidium bromide, while the nonconserved residue (F322), which is also present in P2X4 receptor, responds with a 10-fold higher sensitivity to BzATP, much slower deactivation kinetics, and a higher propensity to form the large dye-permeable pore. We examined the membrane expression of conserved mutants and found that Y51, Q52, G323, and F328 play a role in the trafficking of the receptor to the plasma membrane, while K49 controls receptor responsiveness to agonists. Finally, we studied the importance of the physicochemical properties of these residues and observed that the K49R, F322Y, F322W, and F322L mutants significantly reversed the receptor function, indicating that positively charged and large hydrophobic residues are important at positions 49 and 322, respectively. These results show that clusters of conserved residues above the transmembrane domain 1 (K49–Y51–Q52) and transmembrane domain 2 (G326–K327–F328) are important for receptor structure, membrane expression, and channel gating and that the nonconserved residue (F322) at the top of the extracellular vestibule is involved in hydrophobic inter-subunit interaction which stabilizes the closed state of the P2X7 receptor channel.

## 1. Introduction

The purinergic P2X receptors (P2X1–7) are ATP-gated cation channels that have a widespread distribution in neuronal and non-neuronal cells and are involved in many physiological and pathophysiological processes [1,2,3,4,5,6]. P2X7 is a member of the P2X family [7] with specific hallmark features that forms a large pore after activation with extracellular agonists [8] and has also been shown to induce a variety of cellular responses that are not directly associated with ion channel functions, such as changes in the activation of lipases, kinases, and transcription factors [1,9]. P2X7 is expressed in immune cells, particularly in cells of myeloid lineage [10], astrocytes, and microglia, but the expression of P2X7 in neurons is still not clear [11,12,13]. P2X7 is involved in proinflammatory signaling [14] and apoptosis and plays a pivotal role in a variety of central nervous system (CNS) pathologies such as epilepsy [15] and neuroinflammation [16]. P2X7 also regulates the pathophysiology of psychiatric disorders, including mood disorders [17] and affective disorders such as depression, bipolar disorders, and schizophrenia [18]. Total P2X7 depletion has beneficial effects on social behavior [19]. Genetic polymorphisms have been shown to impact post-transplant outcomes of allogeneic stem cell transplantation [20]. Given its proinflammatory function, P2X7 has been proposed as a therapeutic target for various inflammatory and neurological disorders [10,21,22,23] as well as for autism-like behavior [24,25].

All P2Xs are organized as trimers [26]. Each subunit is composed of two transmembrane domains (TM1 and TM2), an extracellular domain with an agonist-binding site, and cytoplasmic N- and C-terminal domains [27,28]. In response to the binding of extracellular ligands, the P2X7 channel opens to allow the permeation of small cations, such as Na^+^, K^+^, and Ca^2+^ ions, as well as larger organic molecules, such as N-methyl-d-glucamine (NMDG^+^) [29,30]. Functional responses to extracellular ligands characteristic for P2X7 responses also include entry of the fluorescent dye ethidium bromide (EtBr) through a cation-selective channel/pore [29] that is lined by TM2 helices [31,32]. The extracellular vestibule was discovered for the first time in the crystal structure of zebrafish P2X4 (zfP2X4) [28] and confirmed in panda P2X7 [33]. The extracellular vestibule connects the transmembrane domain with the agonist-binding domain and represents a structure that is composed of random coils, β-sheets, and extracellularly oriented parts of the TM1 and TM2 helices [34]. Several functional studies on P2X2 and P2X4 have addressed the role of the extracellular vestibule in receptor function using cysteine mutants and modifications of introduced residues by methanethiosulfonate reagents or cadmium ion accessibility method [34,35,36,37]. The main focus in these studies was on charged residues (E51, E56, and D58, P2X4 numbering) proximal to TM1 that line the lateral portals of the closed human P2X4 receptor. These studies indicate that ions access and enter the pore through lateral fenestrations of the extracellular vestibule, with negatively charged side chains of residues E56 and D58 being important for forming an access pathway for cations [37], and residue E51 contributes to the high Ca^2+^ permeability of P2X4 [35,36,38]. Analysis of alanine-substituted mutants of the extracellular peptide segment proximal to TM2 (the G316-I333 region of rat P2X4) also showed that conserved polar amino acids in this region are important for signal transduction between the ATP-binding domain and the channel gate [39]. Previously, we used alanine- and cysteine-scanning mutagenesis to show that a majority (53%) of the individual substitutions in the extracellular vestibule of rat P2X4 (regions V47-V61 and F324-N338) do not significantly affect receptor function and that five substitutions, namely V49, Y54, Q55, F324, and G325, are critical for P2X4 function [40]. These residues are also present in P2X7 (except V49), and Y51, Q52, and G323 are fully conserved among the mammalian P2X family.

Here, we tested a hypothesis that the conserved residues in the extracellular vestibule and the nonconserved residue F322 (equivalent to F324 in P2X4) are important for P2X7 function. We substituted these residues with an alanine residue and characterized the functional properties of these mutations after expression in human embryonic kidney 293T (HEK293T) cells. The results show that clusters of conserved residues proximal to TM1 and TM2 are important for receptor trafficking, structure, and gating and that P2X7 function is critically dependent on the properties of the nonconserved residue F322 at the top of the extracellular vestibule and between the extracellular and central vestibules.

## 2. Results

### 2.1. Electrophysiological Characterization of Alanine Mutants of Conserved and Unique Amino Acid Residues in the Extracellular Vestibule of Rat P2X7

**Figure 1:**
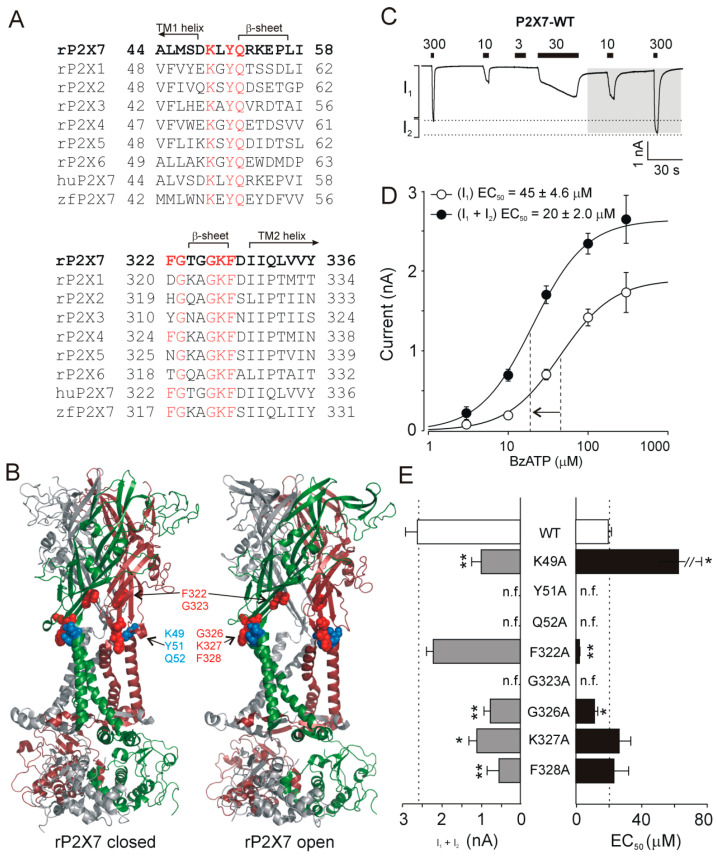
Effect of alanine point mutations of conserved and unique residues in the extracellular vestibule on rat P2X7 (rP2X7) activity. (**A**) Alignment of amino acid sequences A44–I58 and F322–Y336 (rP2X7 numbering) forming extracellular vestibules in seven rat P2X receptors (rP2X1–7), the human P2X7 receptor (huP2X7), and the zebrafish P2X7 receptor (zfP2X7). These regions are composed of extracellularly oriented parts of transmembrane domains (TM1and TM2) α-helices, random coils, and β-sheets. Conserved and unique residues examined in this study are shown in red. (**B**) The topology of conserved and unique residues in the extracellular vestibule of rP2X7 in the apo-closed state (left) and ATP-bound open state (right). The three subunits of the trimeric receptor are shown in green, purple, and gray, and the mutated residues adjacent to the TM1 and TM2 helices are shown as blue and red spheres, respectively. The figure was generated using models of full-length rP2X7 in the apo-closed (PDB ID: 6U9V) and ATP-bound open (PDB ID: 6U9W) states [31] and PyMOL v0.99 (http://www.pymol.org). (**C**) Example recording showing the responses of human embryonic kidney 293T (HEK293T) cells expressing rP2X7 to stimulation with several concentrations (3, 10, 30, and 300 µM) of 2′,3′-O-(benzoyl-4-benzoyl)-ATP (BzATP). An initial application of BzATP evoked a current of lower amplitude, I_1_, and I_2_ is the contribution of the secondary current growth produced by prolonged (40–60 s) exposure to the agonist. In this and the following figures, the horizontal bars indicate the duration of the BzATP application, and gray areas indicate measurements that were used for construction of concentration–response curves and evaluation of EC_50_ after current facilitation. (**D**) The concentration–response curves for the initial (I_1_) and facilitated (I_1_ + I_2_) currents of rP2X7 wild-type (WT). The vertical dashed lines represent the EC_50_ values, and the arrow indicates the leftward shift in the EC_50_ value after current facilitation. The data are presented as the means ± SEMs of 5–35 cells per concentration. All concentration–response curves shown in this and the following figures were generated using a Hill coefficient of 1.3 obtained by fitting the WT receptor. The numbers represent the mean EC_50_ values. (**E**) Alanine mutagenesis of conserved and unique residues in the extracellular vestibule of rP2X7. The maximum amplitude of currents (I_1_ + I_2_) induced by 300 µM BzATP (left); BzATP EC_50_ values for WT and mutant P2X7 receptors evaluated after prolonged exposure to the agonist (right). The mean ± SEM from 5–18 measurements per mutant is shown; * *p* < 0.05 and ** *p* < 0.01, significant differences between WT and alanine mutant; n.f., nonfunctional.

The extracellular vestibule of rat P2X7 (rP2X7) is formed by segments A44–I58 and F322–Y336 that are proximal to TM1 and TM2, respectively (Figure 1A). Structurally, these regions are composed of extracellularly oriented parts of TM1 and TM2 α-helices, random coils, and β-sheets. We examined the functional importance of the fully conserved residues K49, Y51, Q52, G323, G326, K327, and F328, that are present in all P2X1-7 subtypes, and the nonconserved residue F322, which is also present in P2X4. Figure 1B shows the localization of these residues in the extracellular vestibule of rP2X7 receptor.

The wild-type (WT) rP2X7 and alanine mutants of these residues were expressed in HEK293T cells, and the receptor function was examined using patch-clamp whole-cell recordings and dye uptake measurements. We used different concentrations of 2′,3′-O-(benzoyl-4-benzoyl)-ATP (BzATP; 3, 10, 30, 100, or 300 µM) to investigate the effect of mutagenesis on receptor activity and sensitivity to agonists (Figure 1C).

The activation kinetics of P2X7 are known to speed up during repeated [29] or prolonged applications of agonists [41,42,43,44,45,46] in a process known as current facilitation. Consequently, the amplitude of the BzATP-induced current is not stable and increases with recording time. To ensure reproducible responses, each receptor was characterized by the amplitude of the initial current induced by application of 300 µM BzATP to the naïve cell (I_1_), the secondary current growth (I_2_) produced by prolonged (40–60 s) exposure to the agonist, and the amplitude of the maximum current (I_1_ + I_2_) induced by application of 300 µM BzATP after current facilitation (Figure 1C). The EC_50_ value, the agonist concentration producing 50% of the maximal response, was also obtained from concentration–response curves before and after current facilitation (Figure 1D). The results of these investigations are summarized in Table 1 and Figure 1E.

In the WT receptor, the amplitude of responses to 300 µM BzATP increased significantly (*p* < 0.01) on average by 1.5 ± 0.2-fold (I_1_ = 1.7 ± 0.3 nA; I_1_ + I_2_ = 2.6 ± 0.3 nA) after prolonged exposure to the agonist, and the EC_50_ decreased significantly (*p* < 0.01) from 45 ± 4.6 µM to 20 ± 2 µM (Figure 1D). The Y51A, Q52A, and G323A substitutions resulted in nonfunctional receptors that did not respond to 300 µM BzATP, and their EC_50_ values could not be determined. Mutants that exhibited significantly (*p* < 0.01 or *p* < 0.05) reduced maximum responses were K49A, G326A, K327A, and F328A (Figure 1E, left), and the K49A and G326A mutants also showed significant (*p* < 0.05) changes in EC_50_ values compared to that of the WT receptor (WT, EC_50_ = 20 ± 2 µM; K49A, EC_50_ = 63 ± 26 µM; G326A, EC_50_ = 11.6 ± 2.0 µM; Figure 1E, right). Compared to that of the WT receptor, the amplitude of maximum response (I_1_ + I_2_) was not significantly different for the F322A mutant; however, this receptor showed significantly (*p* < 0.01) increased I_1_ (Table 1) and a dramatic (*p* < 0.01) decrease in the EC_50_ value (WT, EC_50_ = 20 ± 2 µM; F322A, EC_50_ = 2.4 ± 0.3 µM; Figure 1E, right). 

These results indicated that all conserved extracellular vestibule residues are important for P2X7 function that and the nonconserved residue F322 may substantially contribute to the gating properties of the channel.

### 2.2. Membrane Expression of Nonfunctional and Low-Functioning Mutants

**Figure 2:**
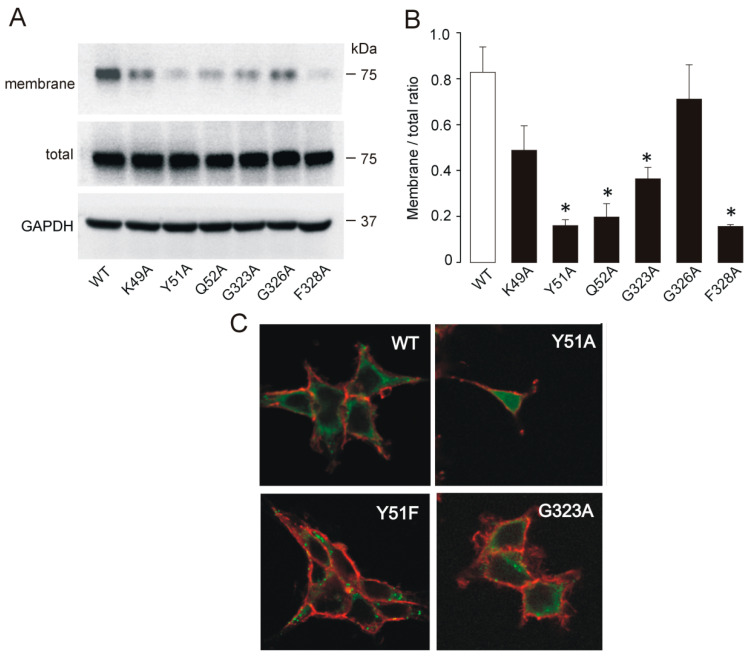
Membrane expression of the P2X7 WT and low-functioning extracellular vestibule mutants. (**A**) Western blots showing the expression pattern of the WT, K49A, Y51A, Q52A, G323A, G326A, and F328A receptors. The expression level of the receptors in the plasma membrane, the total expression level of the receptors, and the expression level of the housekeeping gene GAPDH were examined. (**B**) The relative surface protein expression of the WT and mutant receptors calculated as the ratio of signal intensity of P2X7 expressed at the membrane to the total P2X7 (membrane/total ratio) using GAPDH as a loading control for both the membrane and the total protein expression. The expression level was examined in 3–9 experiments, and the data are expressed as the means ± SEMs; * *p* < 0.05 between WT and alanine mutants. (**C**) Confocal microscopy showing the presence of the WT-YFP, Y51A-YFP, Y51F-YFP, and G323A-YFP tagged receptors (green signal) on the cell surface (red signal; wheat germ agglutinin marked outer membrane) and in the cytosol of transfected HEK293T cells; objective 63x.

In general, the loss of receptor function by substituting the conserved residues with an alanine residue could reflect the altered trafficking of mutants to the plasma membrane or the loss of responsiveness to agonists. We performed quantitative Western blot analysis of the membrane expression of receptors that exhibited a significantly (*p* < 0.01) reduced amplitude of BzATP-induced current (K49A, Y51A, Q52A, G323A, G326A, and F328A) to test a hypothesis that this effect reflects changes in the level of receptors on the cell surface. We examined membrane fractions of total protein derived from HEK293T cells expressing the WT or a mutant receptor (Figure 2A). Compared to the expression of the WT receptor, the Y51A, Q52A, G323A, and F328A mutants showed significantly (*p* < 0.05) reduced expression in the plasma membrane fraction, whereas other mutants (K49A and G326A) were present in variable quantities (Figure 2B). 

We also examined membrane expression of the WT, Y51A, and G323A receptors tagged with yellow fluorescent protein (YFP) using confocal microscopy (Figure 2C). These experiments showed that WT-YFP was present on the cell surface, while the Y51A-YFP mutant was retained in the cytosol, and the G323A-YFP mutant was partly in the membrane and partly retained in the cytosol.

Taken together, these results suggest that for the Y51A, Q52A, and F328A mutants, the loss of receptor function reflects the lack of their expression at the plasma membrane, while altered function of K49A, G326A, and possibly G323A is due to changes in the intrinsic channel properties.

### 2.3. Rescue of Receptor Function by Replacing K49 with a Positively Charged Residue

**Figure 3:**
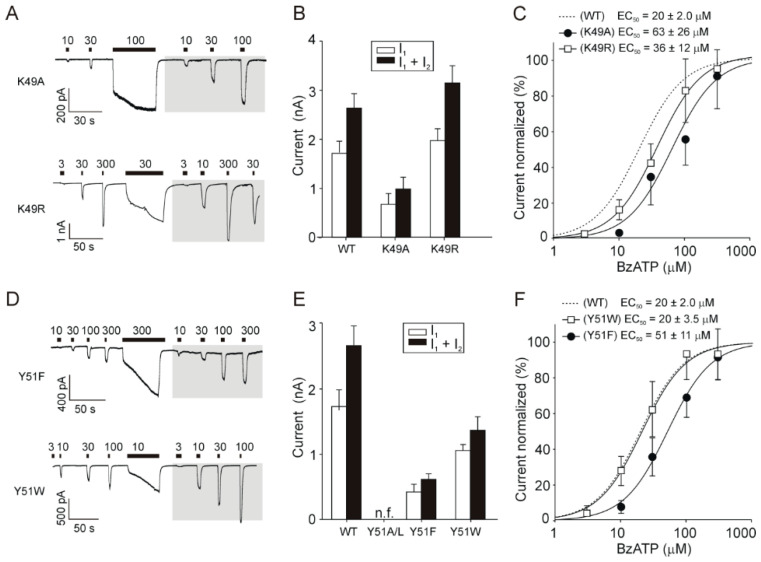
Rescue of receptor function by replacing K49 with an arginine and Y51 with an aromatic residue. (**A**) Example records of BzATP-induced responses mediated by the K49A and K49R receptors. (**B**) The amplitude of 300 µM BzATP-induced initial (I_1_) and maximum (I_1_ + I_2_) currents for the WT, K49A, and K49R receptors. (**C**) The concentration–response curves for WT (dotted curve), K49A, and K49R. (**D**) Example records of BzATP-induced responses mediated by the Y51F and Y51W receptors. (**E**) The amplitude of 300 µM BzATP-induced initial (I_1_) and maximum (I_1_ + I_2_) currents for the WT, Y51F, and Y51W receptors. The Y51A and Y51L mutations were nonfunctional (n.f.). (**F**) The concentration–response curves for the WT (dotted curve), Y51F, and Y51W evaluated after prolonged exposure to the agonist. The data are presented as the means ± SEMs of 5–35 cells per concentration, and the numbers represent the mean of EC_50_ values after current facilitation.

In contrast to the K49A mutant, the K49R mutant (Figure 3A) did not significantly alter the maximum amplitude of the BzATP-induced current compared with that of the WT receptor (WT, I_1_ + I_2_ = 2.6 ± 0.3 nA; K49A, I_1_ + I_2_ = 1.0 ± 0.2 nA; *p* < 0.01; K49R, I_1_ + I_2_ = 3.1 ± 0.4 nA; *p* > 0.05; Figure 3B), and the K49R receptor sensitivity to the agonist was also comparable with that of the WT receptor (WT, EC_50_ = 20 ± 2 µM; K49A, EC_50_ = 63 ± 26 µM; *p* < 0.05; K49R, EC_50_ = 36.2 ± 12 µM; *p* > 0.05; Figure 3C).

These data showed that the positive charge at position 49 is important for P2X7 channel gating.

### 2.4. Rescue of the Receptor Function by Replacing Y51 with Aromatic Residues

We examined the importance of the physicochemical properties of various amino acid residues substituted at the Y51 position to further determine the P2X7 specificity that is regulated by this residue. The Y51L substitution generated a nonfunctional receptor. Next, we examined substituting this residue with phenylalanine and tryptophan, which are nonpolar aromatic residues that differ in volume. The Y51F substitution generated a receptor that was functional (Figure 3D) and expressed in the plasma membrane (Figure 2C), but it exhibited significantly (*p* < 0.01) reduced maximum current amplitude (Figure 3E) and sensitivity to the agonist (Figure 3F). The Y51W substitution recovered the receptor activity (WT, I_1_ + I_2_ = 2.6 ± 0.3 nA; Y51F, I_1_ + I_2_ = 0.6 ± 0.1 nA; *p* < 0.01; Y51W, I_1_ + I_2_ = 1.4 ± 0.2 nA; *p* < 0.05; Figure 3 E) and sensitivity to the agonist (WT, EC_50_ = 20 ± 2 µM; Y51F, EC_50_ = 51.2 ± 11 µM; *p* < 0.05; Y51W, EC_50_ = 20.3 ± 3.5 µM; Figure 3F) much better than the Y51F substitution.

We did not attempt to replace Q52 with another residue to rescue the receptor function because we have previously shown that introducing any amino acids of similar structure at an equivalent position in P2X4 (N, E, T, or K) could not rescue the receptor function [40].

These results show that a large aromatic residue at position 51 is essential for receptor trafficking and function.

### 2.5. Recovery of Normal Receptor Function by Replacing F322 with Hydrophobic/Aromatic Residues

**Figure 4:**
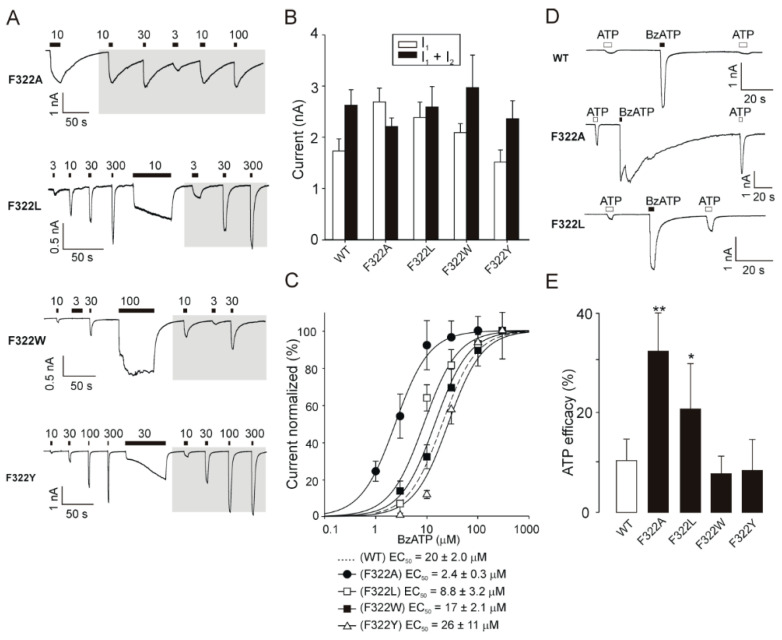
Rescue of receptor function by replacing F322 with a hydrophobic aliphatic or an aromatic residue. (**A**) Example records of BzATP-induced responses mediated by the F322A, F322L, F322W, and F322Y receptors. (**B**) The amplitude of 300 µM BzATP-induced initial (I_1_) and maximum (I_1_ + I_2_) current for WT, F322A, F322L, F322W, and F322W; in the case of F322A, 100 µM BzATP was used. (**C**) The concentration–response curves for the WT (dotted line) and F322 mutants evaluated after prolonged exposure to the agonist. The data are presented as the means ± SEMs of 4–21 cells per concentration. (**D**) Representative recordings of the WT, F322A, and F322L P2X7 currents stimulated by the application of ATP or BzATP (both 100 µM). (**E**) Summarized data showing the efficacy of ATP for the WT, F322A, F322L, F322W, and F322Y receptors. For each of these receptors, the maximum current amplitude stimulated by 100 µM ATP was compared to that stimulated by 100 µM BzATP (ATP efficacy, in %). The data are presented as the means ± SEMs from 5–18 cells per mutant. Statistical significance was estimated by comparing the efficacy between the WT and mutant receptors (** *p* < 0.01; * *p* < 0.05).

The F322A mutant exhibited no changes in I_1_ + I_2_ compared with that of the WT receptor (WT, I_1_ + I_2_ = 2.6 ± 0.3 nA; F322A, I_1_ + I_2_ = 2.2 ± 0.2 nA; *p* > 0.05), but the maximum amplitude of the initial BzATP-induced current in naïve cells was significantly (*p* < 0.05) higher in the F322A receptor than in the WT receptor (WT, I_1_ = 1.7 ± 0.2 nA; F322A, I_1_ = 2.7 ± 0.3 nA; Table 1 and Figure 4A,B), indicating that the activation kinetics of F322A speeds up quickly following stimulation with agonist and that the current quickly reached a steady state (Figure 4A). The deactivation time constant measured in naïve receptors as the decay of current after washout of 3 µM BzATP (τ_off_) dramatically increased by 60-fold compared with that in the WT receptor (WT, τ_off_ = 0.45 ± 0.06 s; F322A, τ_off_ = 27.1 ± 2.7 s; *p* < 0.01; Table 1). This effect was apparently due to the significantly (*p* < 0.01) increased sensitivity of the F322A receptor to agonists (WT, EC_50_ = 20 ± 2 µM; F322A, EC_50_ = 2.4 ± 0.3 µM; Figure 4C).

Mutating F322 to another nonpolar residue (F322L or F322W) or a polar aromatic residue (F322Y) rescued the slow current facilitation and fast deactivation (Figure 4A and Table 1) and recovered the receptor sensitivity to BzATP (WT, EC_50_ = 20 ± 2 µM; F322L, EC_50_ = 8.8 ± 3.2 µM; *p* < 0.05; F322W, EC_50_ = 17 ± 2.1 µM; *p* > 0.05; F322Y, EC_50_ = 26 ± 11 µM; *p* > 0.05; Figure 4C) and the deactivation kinetics (WT, τ_off_ = 0.45 ± 0.06 s; F322L, τ_off_ = 2.19 ± 0.34 s; *p* < 0.01; F322W, τ_off_ = 1.39 ± 0.13 s; *p* < 0.01; F322Y, τ_off_ = 1.47 ± 0.25 s; *p* < 0.01; Figure 4A and Table 1) to various degrees.

rP2X7 WT is characterized by low sensitivity to ATP, and its efficacy is only approximately 10% of that of BzATP when using a concentration of 100 µM of both drugs (Figure 4D,E). For the F322A receptor, the ATP efficacy significantly (*p* < 0.01) increased by approximately 3-fold, and this effect could also be reversed to various degrees by substituting this residue with nonpolar or polar hydrophobic residues (Figure 4D,E).

We also performed substitutions at G323, which is located at the top of the extracellular vestibule near F322, in an attempt to rescue the receptor function. These experiments showed that substitutions with amino acids of similar size (G323S or G323P) did not generate functional receptors (Table 1).

Together, these data indicate that the hydrophobicity and size, rather than aromaticity, of the residue at position 322 are important for P2X7 gating to a closed state. Additionally, these data also show that nearby conserved glycine at position 323 is essential for P2X7 function and probably operates as a hinge when the receptor undergoes activation-induced conformational changes.

### 2.6. EtBr Accumulation by Cells Expressing Extracellular Vestibule Mutants

**Figure 5:**
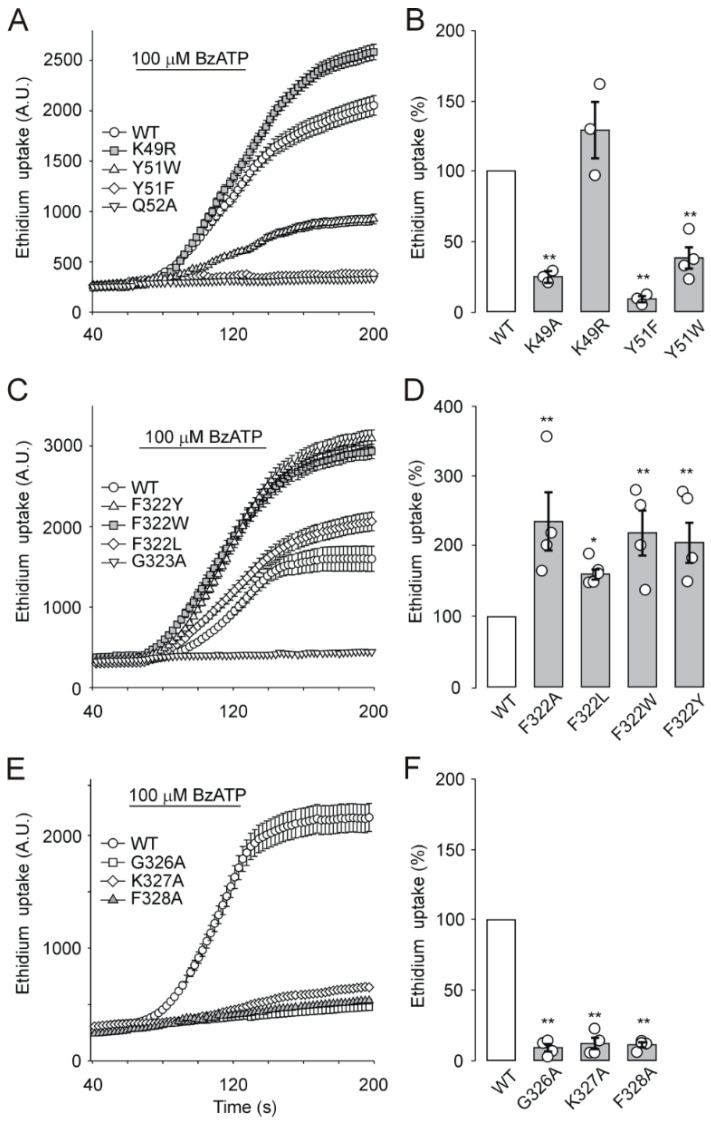
BzATP-stimulated EtBr accumulation by cells expressing the WT and mutant P2X7 receptors. (**A**,**C**,**E**) Example experiment showing fluorescent measurement of EtBr uptake by HEK293T cells expressing WT (open circles) and mutant P2X7 receptors. EtBr accumulation was triggered by 100 µM BzATP (horizontal bar), and dye uptake was determined as the amplitude of the EtBr signal 100 s after the beginning of the BzATP application. Signals from 5–10 cells were averaged, and the data points are the means ± SEMs. The intensity of light emission was measured every 1–2 s; for clarity, some data points have been omitted. A.U., arbitrary units. Example of 3–7 similar experiments. (**B**,**D**,**F**) Summary histograms showing the BzATP-stimulated EtBr uptake (in % of control) by cells expressing the WT receptor (white column) and mutant P2X7 receptors. Data are presented as the means ± SEMs of 3–7 experiments; statistical significance was estimated by using one-way ANOVA followed by post-SNK test; *p* < 0.05 (*) and *p* < 0.01 (**).

We investigated the BzATP-stimulated accumulation of fluorescence dye EtBr by HEK293T cells expressing extracellular vestibule mutants to compare the electrophysiological data with the pore formation properties of mutated receptors. Figure 5A shows that EtBr accumulation by cells expressing the WT receptor increased almost linearly during the first 100 s of the 100 µM BzATP application. In agreement with the patch-clamp studies, BzATP-induced EtBr was not taken up by cells expressing the Y51A, Y51L, Q52A, G323A, G323S, and G323P mutants (Figure 5 and Table 1). Compared to that of the WT receptor, the rate of EtBr accumulation was significantly (*p* < 0.01) reduced by ~90% in cells expressing the K49A, Y51F, Y51W, G326A, K327A. and F328A mutants (Figure 5 and Table 1) and increased (*p* < 0.01) in cells expressing the F322A, F322L, F322W, and F322Y mutants (Figure 5C,D). The accumulation of EtBr by cells expressing the K49R mutant (Figure 5A,B) was comparable with that of the WT receptor.

These data show that dye uptake by cells expressing P2X7 is critically dependent on conserved residues in the extracellular vestibule and is controlled by F322.

## 3. Discussion

We report here the functional characterization of selected extracellular vestibule mutants of rP2X7 that were analyzed for their capacities to induce membrane current, control receptor expression in the membrane, and open a large membrane pore permeable to EtBr. Our results revealed that clusters of conserved residues above the TM1 (K49–Y51–Q52) and TM2 (G326–K327–F328) domains are important for membrane expression, receptor structure, and gating properties and that the nonconserved residue (F322), which is unique for P2X7 and P2X4, dramatically controls the affinity of the receptor for its ligands, deactivation kinetics, and dye uptake ability. 

**Figure 6:**
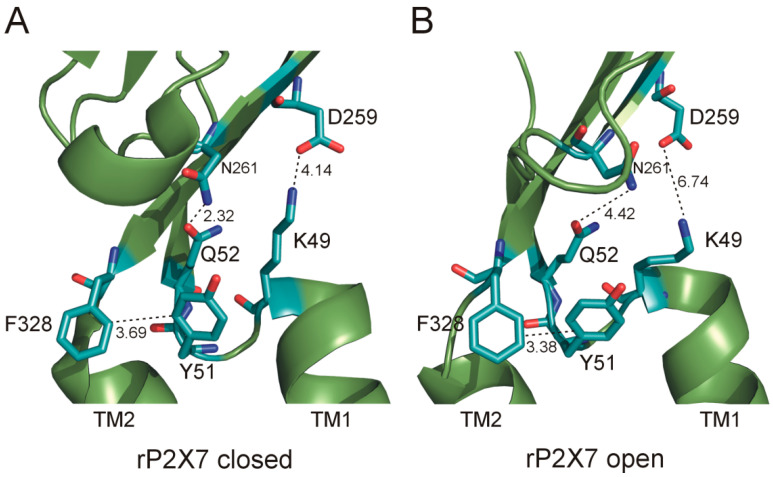
The possible interactions of K49, Y51, and Q52 with other residues in the same rP2X7 subunit. (**A**) Residue K49 can form an electrostatic interaction with D259 (4.14 Å), Y51 can form stacking interactions with F328 (3.69 Å), and Q52 can form an electrostatic interaction with N261 (2.32 Å) in the apo-closed state of rP2X7. (**B**) The interactions of K49–D259 and Q52–N261 are weaker, while the stacking interaction of Y51–F328 is stable in the ATP-bound open state of the rP2X7 channel. The model represents the rP2X7 receptor in the closed (PDB ID: 6U9V) and open (PDB ID: 6U9W) states [31]. This figure was generated using PyMOL v0.99 (http://www.pymol.org). Side chains are represented as sticks, and all hydrogen atoms were omitted for clarity.

We found here that the maximum current amplitude and receptor sensitivity to BzATP were significantly reduced for the K49A mutant compared to those for the WT receptor. In contrast, this mutant at an equivalent position in P2X4 shows no significant difference in the current amplitude or EC_50_ compared to that of the WT receptor [40], indicating that the conserved residue K49 plays a nonconserved role in P2X7. Receptor K49R has back both positive charge and function, and the cryoelectron microscopy (cryo-EM) structures of full-length rP2X7 receptor in apo and ATP-bound states (Figure 6) suggest that K49 is in part on the surface of the receptor, thus allowing electrostatic interactions with adjoining surfaces. K49 possibly interacts via a H-bond with D259 of the same subunit in the closed state of the receptor, and this interaction is weakened after the transition to the ATP-bound open state (the distance between these residues is increased from 4.14 Å in the closed state to 6.74 Å in the open state). This interaction might be important for TM domain structure and conformational changes during channel gating. The Y51A and Q52A mutants are nonfunctional and are not expressed in the membrane. Previous studies performed on P2X2 and P2X4 also showed that alanine or cysteine substitutions of the residues equivalent to Y51 and Q52 do not form functional channels [34,40,47]. Interestingly, alanine or cysteine substitutions of the residue equivalent to Q52 had no effects on ATP sensitivity or peak current amplitude in the P2X1 expressed in oocytes [48,49], suggesting that there are subtype differences in the ability to tolerate this mutation and/or that the expression system might play a role in receptor trafficking. In a study addressing the molecular dynamics simulations of zfP2X4, the Y54 residue was hypothesized to form a H-bond with the D264 residue from the same subunit, implying that this interaction is important for channel opening [50]. Our results seem to support this idea because the P2X7 Y51F mutant, which lacks a hydroxyl group, exhibits a sensitivity to agonists that is significantly lower than that of the WT or Y51W receptor. However, Figure 6 suggests that the interaction between Y51 and N261, which is equivalent to P2X4 D264, might not be very strong (4.34 Å). Our study further shows that stacking interactions are most likely important because an aromatic residue is critical at position 51 for receptor function. The possible partner of Y51 is F328 from the same subunit (Figure 6). In agreement with this assumption, we found that the F328A mutant exhibits reduced membrane expression and maximum current amplitude that are similar to those of the Y51A mutant. The cryo-EM structures of rP2X7 (Figure 6) suggest that the N261 residue could interact with the Q52 residue in the closed state (2.32 Å) and that this interaction is weakened in the open state (4.42 Å). Altogether, our data indicate that the cluster of conserved residues above TM1 interacts with D259/N261 of the same subunit based on H-bond or electrostatic interactions (Figure 6) and that these interactions play a critical role in the mutual axial orientation of the TM1 and TM2 helices to a position that is important for the function of the receptor. Similarly, a strong interaction might exist among the three residues Q52, Y51, and K49 themselves; thus, it is possible that this cluster is so important that it limits proper folding of the protein and consequently its transport to the membrane. It is also possible to conclude that the Y51A and Q52A substitutions cause a similar or even stronger rightward shift in the potency of the agonist for P2X7 than the mutation of the K49 residue.

**Figure 7:**
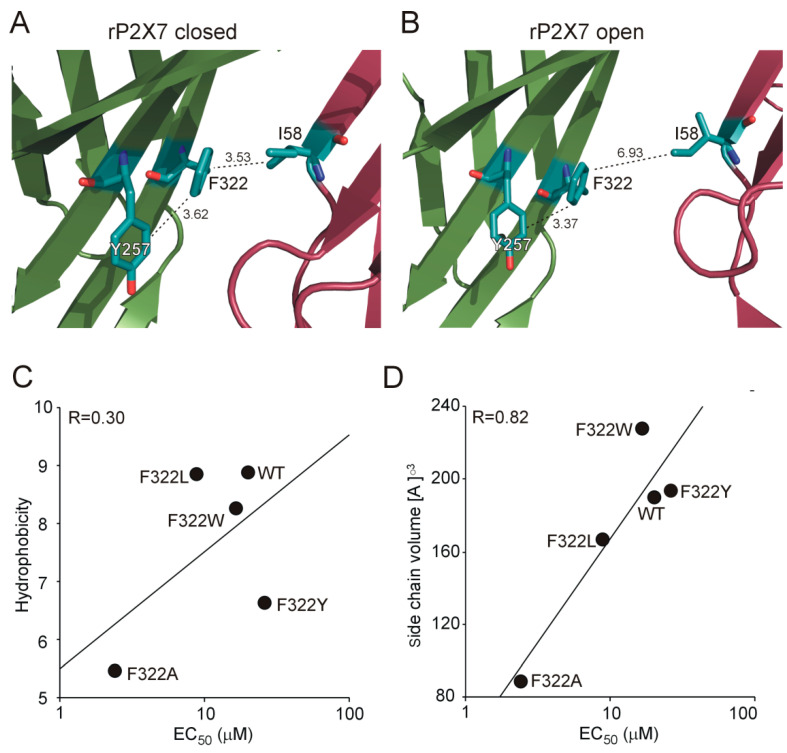
The possible interactions of F322 with other residues in β-sheets of the same or neighboring rP2X7 subunit. (**A**) In the apo-closed state of rP2X7, F322 can interact with Y257 of the same subunit (3.62 Å) or I58 of the neighboring subunit (3.53 Å). (**B**) While the positions of Y257 and F322 are not substantially changed in the ATP-bound open state (3.7 Å), the positions of F322 and I58 are remote in the open state (6.93 Å). (**C**) Correlation between the log EC_50_ values and the hydrophobicity of the amino acid side chain substituent. (**D**) Correlation analysis between the log EC_50_ values and the volume of the amino acid side chain substituent. The model represents the rP2X7 receptor in the closed (PDB ID: 6U9V) and open (PDB ID: 6U9W) states [31].

Substitutions of the nonconserved residue (F322), which is present only in P2X7 and P2X4, at the top of the extracellular vestibule and between the extracellular and central vestibules showed an enhanced facilitation of current kinetics, an accelerated dye uptake, an increased affinity for its ligands, and a slower rate of deactivation, indicating that this residue most likely controls gating and intra-subunit signal transduction from the extracellular agonist-binding site to the transmembrane domain. In contrast, alanine or cysteine substitutions of phenylalanine at equivalent positions in P2X4 respond to ATP with substantially reduced (approximately 10-fold) maximum current amplitude and exhibit only approximately 2-fold higher sensitivity to agonists [40], suggesting that F322 has receptor-specific function in P2X7. The cryo-EM structure of rP2X7 (Figure 7) shows that F322 is located between two potentially interacting residues. F322 could interact through π–π interactions with Y257 of the same subunit, and F322 could interact through hydrophobic interactions also with I58 of the neighboring subunit in the closed state. While the F322–Y257 interaction is relatively stable, the F322–I58 interaction is disrupted after the transition to the open state (Figure 7A,B). This strongly indicates that this inter-subunit interaction could stabilize the closed state. Substitutions of F322 with a hydrophobic residue recovers the receptor function to various degrees (F322A << F322L < F322W ≤ F322Y < WT). Correlation analysis between the EC_50_ values and the hydrophobicity (Figure 7C) or volume (Figure 7D) of the amino acid side chain substituent revealed that the volume of the hydrophobic residue is important, which could explain why substitution with alanine residue is so efficient. We assume that leucine is still able to interact with the counteracting isoleucine, but alanine is too short. This analysis also suggests that substitution with nonaromatic aliphatic amino acids apparently causes movement and higher mobility of the protein between subunits in the closed state, which might weaken inter-subunit interactions. Tyrosine or tryptophan could interact more strongly with β-sheets of their own subunit, which might also cause weakening of potential interactions with neighboring subunits in the closed state.

Although the nearby G323 residue is conserved among the P2X receptor subtypes, its critical functional importance is shown only for P2X4 [40] and P2X7 (here). In P2X1 [51,52] and P2X2 [34,53], a cysteine substitution of the glycine residue at the equivalent positions is functional. It is thus possible that the function of G323 is related to the proximity of F322. Both residues are located on a long β-sheet connecting the agonist-binding site with the TM2 domain (from M304 to F328). We speculate that G323 probably operates as a hinge which might be crucial for the channel to be functional in either conformational stage: for twisting of the β-sheet and extracellular widening after receptor activation in the open state and for inter-subunit interaction mediated by F322 in the closed state. 

The G326A, K327A, and F328A mutants exhibited significantly reduced (by ~60–80%) amplitudes of agonist-evoked currents without changes in receptor sensitivity to agonists, except G326A, which showed slightly enhanced (*p* < 0.05) sensitivity to agonists. The dye uptake ability of cells expressing these mutants being inhibited by ~90% thus must be due to the reduced density of receptors in the membrane, which was confirmed for the F328A mutant. Our results show that both reduced membrane expression (Y51A, Q52A, G323A, and F328A) and lower receptor sensitivity to agonists (K49A and Y51F) abolish dye uptake ability, while increased receptor sensitivity (F322 mutants) results in accelerated dye uptake by cells expressing these mutant receptors. Overall, the results of previous studies [29,31,32,41,42,43,44,45,46] and our study suggest that the mechanisms of dye uptake are complex, and the possibility that G326A and K327A show reduced ability to form a large pore needs further investigation.

In conclusion, these results identify a previously unknown outcome of mutagenesis of the extracellular vestibule in the rP2X7 receptor. The clusters of conserved residues above TM1 (Y51–Q52) and TM2 (G326–K327–F328) are important for receptor trafficking and structure and thus have functions similar to those observed previously in the P2X4 receptor [40]. The most significant difference was found in the function of K49 and F322 residues, which control receptor channel gating in a P2X7-specific manner. The results reveal that the portals of the extracellular vestibule, which have been described previously as a pathway for ions to access and enter the transmembrane pore [34,35,36,37,38], also play a role in P2X7 channel gating.

## 4. Materials and Methods

### 4.1. Cell Culture and Transfection

Experiments were performed using HEK293T cells (ATCC, Manassas, VA, USA), which were grown in Dulbecco’s modified Eagle’s medium (Gibco, Rockville, MD, USA) supplemented with 10% fetal calf serum (ATCC, Manassas, VA, USA), 50 U/mL penicillin, and 50 μg/mL streptomycin (Sigma, St Louis, MO, USA) in a humidified 5% CO_2_ atmosphere at 37 °C. The cells were cultured in 75 cm^2^ plastic culture flasks (NUNC, Rochester, NY, USA) for 36–72 h until they reached 80–95% confluence. On the day before transfection, ~150,000 cells were seeded on 35 mm culture dishes (Sarstedt, Newton, NC, USA) and incubated at 37 °C for at least 24 h. Transfection was performed using 2 μg of DNA and 2 μL of jetPRIME reagent in 2 mL of Dulbecco’s modified Eagle’s medium according to the manufacturer’s instructions (PolyPlus-transfection, Illkirch, France). After 24 h of incubation, the transfected cells were mechanically dispersed and reseeded on 35 mm Corning 3294 CellBIND Surface cell culture dishes (Corning, City of Corning, NY, USA) for 2–8 h prior to recording. For the dye uptake measurements, the transfected cells were plated on 12 mm poly-L-lysine-coated coverslips (Glaswarenfabrik Karl Hecht KG, Sandheim, Germany).

### 4.2. DNA Constructs

Rat full-length rP2X7 receptor cDNA subcloned into the biscistronic enhanced fluorescent protein expression vector pIRES2-EGFP was a kind gift from Dr. S.S. Stojilkovic, NICHD/NIH, Bethesda, MD, USA. For constructing the YFP C-terminal-tagged rP2X7, full-length rP2X7 was first amplified by PCR using a 3′ oligonucleotide primer (Eurofins) designed to replace the stop codon by an in Frame Xho1 restriction site as previously described for other P2X subtypes [54,55]. The full-length rP2X7 was then subcloned into pcDNA3 containing an Xho1–Xba1 cassette coding for YFP. Single-point mutant receptors were constructed by PCR amplification using specific overlapping oligonucleotide primers (synthesized by Eurofins CZ s.r.o., Prague, Czechia) and a QuikChange II site-directed mutagenesis kit (Stratagene, La Jolla, CA, USA). All of the constructs were verified by sequencing (ABI PRISM 3100, Applied Biosystems, Foster City, CA, USA) performed by SeqMe (Dobříš, Czechia).

### 4.3. Whole-Cell Patch-Clamp Recording

Currents were recorded in a whole-cell configuration using an Axopatch 200B patch-clamp amplifier (Axon Instruments, Union City, CA, USA). All current recordings were captured and stored using the pClamp 9.0 software package in conjunction with the Digidata 1322A A/D converter (Molecular Devices, Silicon Valley, CA, USA). The experiments were performed on single cells with an average capacitance of 10 pF, and the membrane potential was held at –60 mV. Patch electrodes were filled with solution containing 145 mM NaCl, 10 mM EGTA, and 10 mM HEPES; the pH was adjusted using 10 M NaOH to 7.3 [46]. The osmolarity of this solution was 293 mOsm. During the experiments, the cells were continuously perfused with an extracellular solution containing 142 mM NaCl, 3 mM KCl, 2 mM CaCl_2_, 1 mM MgCl_2_, 10 mM HEPES, and 10 mM D-glucose and adjusted to pH 7.3 using 1 M NaOH (Sigma, St Louis, MO, USA). All solutions with agonists were prepared on the day of experiment and were applied using the RSC-200 Rapid Solution Changer (Biologic, Claix, France). Sensitivity of mutated receptors was measured using a short (2–5 s) application of 3–300 µM 2′,3′-O-(benzoyl-4-benzoyl)-ATP (BzATP; Sigma, St Louis, MO, USA), an artificial but potent agonist of the P2X7 receptor [27]. To estimate the EC_50_ values of naïve (not previously stimulated) cells, the responses from 15–50 different naïve cells were pooled, and dose–response curves were constructed using the mean values.

### 4.4. Ethidium Bromide Uptake 

Studies on the cellular accumulation of EtBr were performed using an epifluorescent microscope (Olympus BX50WI, Melville, NY, USA). Transfected HEK-293T cells plated on glass coverslips were bathed in normal extracellular solution, imaged using the 40× water immersion objective at room temperature (20–25 °C), and identified by the fluorescence signal of green fluorescein protein. EtBr (5 μM) and BzATP (100 μM) in normal extracellular solution were added using a fast gravity-driven microperfusion system made in our laboratory and consisting of nine glass tubes, each approximately 400 μm in diameter, with a common outlet of approximately 300 μm in diameter. The application tip was routinely positioned at about a 500 μm distance from the recorded cells and approximately 50 μm above the surface of the coverslip. Change in fluorescence was recorded using MicroMAX CCD camera (Princeton Instruments, Roper Scientific GmbH, Martinsried, Germany). Hardware control and image analysis were performed using MetaFluor software (Molecular Devices, Downingtown, PA, USA). EtBr was excited at 526 nm, and emission light was collected at 605 nm.. The average fluorescence signal of 10 cells was analyzed on each coverslip and each experiment was repeated 3–7 times. The rate of dye uptake was determined as the amplitude of EtBr signal after 100 s of agonist application.

### 4.5. Cell-Surface Biotinylation Assay 

Cell-surface proteins were biotinylated using EZ-Link sulfosuccinimidyl-2-(biotinamido) ethyl-1,3-dithio-propionate (EZ-link sulfo-NHS-SS-biotin, Pierce, Rockford, IL, USA) following established protocol [56,57]. Briefly, HEK293T cells expressing WT or mutant forms of rP2X7 were washed (3 times) with ice-cold phosphate-buffered saline (PBS) (4 °C) and incubated with sulfo-NHS-SS-biotin solution (1 mg/mL) prepared in PBS, pH 8.2, supplemented with 0.1 mM CaCl_2_ and 1 mM MgCl_2_ for 1 h at 4 °C. Nonreacted sulfo-NHS-SS-biotin was quenched by washing cells with cold PBS, pH 8.2, containing 100 mM glycine, 0.1 mM CaCl_2_, and 1 mM MgCl_2_. Cells were harvested in lysis buffer (50 mM Tris-HCl, pH 7.5, 150 mM NaCl, 1 mM EDTA, 0.1% (*v*/*v*) Nonidet P-40, 1% (*v*/*v*) SDS and protease inhibitor (Complete Mini EDTA-free, Roche Molecular Biochemicals, Indianapolis, IN, USA)) before gentle sonication and centrifugation at 14,000× *g* for 10 min at 4 °C. Biotinylated proteins were pulled down from equivalent amounts (~1 mg) of “total” protein (whole cell lysate) with 50 µL of neutravidin agarose beads (Thermo Fisher Scientific, Pierce protein biology products, Rockford, IL, USA) and eluted into 50 µL of Laemmli sodium dodecyl sulfate–polyacrylamide gel electrophoresis sample buffer supplemented with 50 mM dithiothreitol. To determine the amounts of biotinylated P2X7 (membrane) or “total” P2X7 and glyceraldehyde-3-phosphate dehydrogenase (GAPDH) expression, 5 µL of eluted protein and equivalent amounts (~50 μg) of “total” protein were separated on 4–15% precast gels (Bio-Rad Laboratories, Hercules, CA, USA), followed by Western blot with anti-P2X7 monoclonal (Alomone Lab Ltd. Har Hotzvim Hi-Tech Park, Jerusalem, Israel) or GAPDH (Sigma, St Louis, MO, USA), antibody, and detected using horseradish peroxidase coupled anti-rabbit or anti-goat Fc-IgG antibody at dilutions of 1:1000 and 1:10,000, respectively. The chemiluminescent signals were captured using an Image Analyzer (LAS-1000 plus; Fuji Photo Film Co., Ltd. Minato-ku, Tokyo, Japan), and signal intensity (arbitrary densitometric units) was quantified using Image J software (National Institutes of Health, free public access). The relative surface P2X7 expression was calculated as the ratio of membrane to the total P2X7 protein after normalization against GAPDH.

### 4.6. Membrane Expression of YFP-Tagged Receptors

Transiently transfected HEK293T cells with YFP-tagged rP2X7-WT receptor and its mutants (Y51A-YFPtg, Y51F-YFPtg, and G323A-YFPtg) were plated onto 12 mm poly-L-lysine (Sigma, St Louis, MO, USA)-coated coverslips and cultured in Opti-MEM (Invitrogen, Carlsbad, CA, USA) supplemented with 5% fetal calf serum in a water-saturated atmosphere of 5% CO2 and 95% air at 37 °C. The following day, cells were fixed in ice-cold 4% formaldehyde, pH 7.4, in phosphate-buffered saline for 15 min and marked by wheat germ agglutinin conjugated with Alexa633 (6 μL in 4 mL, 10 min, RT). After washing residual agglutinin by PBS, coverslips were mounted in Aqua/Poly-Mount medium (Polysciences, Warrington, PA, USA) onto glass slides. The localization of YFP-tagged receptors in cells was examined by laser scanning confocal microscopy (Leica TCS SP2 AOBS, Leica Microsystems CMS GmbH, Mannheim, Germany). Images were collected under a 63× objective lens with further zoom applied.

### 4.7. Calculations 

The concentration–response data points were fitted with the equation y = I_max_/(1 + (EC_50_/x)^h^), where y is the amplitude of the current evoked by BzATP, I_max_ is the maximum current amplitude induced by 300 μM BzATP, EC_50_ is the agonist concentration producing 50% of the maximal response, h is the Hill coefficient, and x is the concentration of BzATP (SigmaPlot 2000 v9.01; SPSS Inc., Chicago, IL, USA). Hill coefficient was fixed to 1.3 in all experiments, and a value obtained for the P2X7 WT by fitting. The kinetics of deactivation (current decay evoked by washout of 30 or 100 μM BzATP in naïve (not previously stimulated) cells) were fitted by a single exponential function (y = A_1_ exp(−t/τ)) or by the sum of two exponentials (y = A_1_ exp(−t/τ1) + A_2_ exp(−t/τ2)) using the program pClamp 10 (Molecular Devices), where A_1_ and A_2_ are relative amplitudes of the first and second exponentials, and τ1 and τ2 are the time constants. Weight time constant was calculated as y = ((A_1_τ_1_) + (A_2_τ_2_))/(A_1_+A_2_). The derived time constants for deactivation were labeled as τ_off_.

### 4.8. Statistical Analysis

All numerical values in the text are reported as means ± SEMs. Comparisons between two groups were performed by Student’s t-test. Statistical comparison of multiple groups was made by using one-way ANOVA followed by SNK or Dunnett’s test (** *p* < 0.01 and * *p* < 0.05) in SigmaStat 2000 v9.0, for comparison to a single control.

## Figures and Tables

**Table 1:** Characterization of single-point vestibule mutants of rat P2X7 (rP2X7).

rP2X7	I_1_ (nA)	I_1_ + I_2_ (nA)	(I_1_) EC_50_ (µM)	(I_1_ + I_2_) EC_50_ (µM)	τ_off_ (s)	EtBr Uptake (%)
WT	1.7 ± 0.2	2.6 ± 0.3	45.2 ± 4.6	20.9 ± 2.5	0.45 ± 0.06	100 ± 5
K49A	0.7 ± 0.2 **	1.0 ± 0.2 **	92 ± 28 *	63 ± 26 *	0.25 ± 0.41	25 ± 4 **
K49R	1.9 ± 0.2	3.1 ± 0.4	70.9 ± 10.3	36.2 ± 12	0.62 ± 0.10	129 ± 22
Y51A/L	n.f.	n.d.	n.d.	n.d.	n.d.	n.d.
Y51F	0.4 ± 0.1 **	0.6 ± 0.1 **	80.6 ± 19 *	51.2 ± 11 *	0.72 ± 0.27	9.2 ± 2 **
Y51W	1.0 ± 0.1 *	1.4 ± 0.2 *	30.8 ± 2.8	20.3 ± 3.5	0.84 ± 0.22	38 ± 7.5 **
Q52A	n.f.	n.d.	n.d.	n.d.	n.d.	n.d.
F322A	2.7 ± 0.3 *	2.2 ± 0.2	8.6 ± 3.4 **	2.4 ± 0.3 **	27.1 ± 2.7 **	235 ± 41 **
F322W	2.1 ± 0.2	3.0 ± 0.6	35.5 ± 5.3	16.5 ± 2.1	1.39 ± 0.13 **	219 ± 31 **
F322Y	1.5 ± 0.2	2.3 ± 0.4	57.0 ± 27.2	26 ± 10.9	1.47 ± 0.25 **	205 ± 28 **
F322L	2.4 ± 0.3 *	2.6 ± 0.4	12.7 ± 8.1 **	8.8 ± 3.2 **	2.19 ± 0.34 **	160 ± 6 *
G323A/S/P	n.f.	n.d.	n.d.	n.d.	n.d.	n.d.
G326A	0.5 ± 0.1 **	0.8 ± 0.2 **	25.8 ± 4.5 *	11.6 ±2.0 *	1.28 ± 0.15 **	9 ± 3 **
K327A	0.8 ± 0.3 *	1.1 ± 0.2 *	53.4 ± 11.8	26.7 ± 7.1	0.58 ± 0.09	12 ± 4 **
F328A	0.3 ± 0.1 **	0.5 ± 0.3 **	46.0 ± 25	23.5 ±5.2	0.23 ± 0.06	11 ± 2 **

Each receptor was examined for its initial current (I_1_) and secondary current growth (I_2_) when stimulated with 300 µM BzATP. The sensitivity to agonist was examined in naïve, not previously stimulated, cells ((I_1_) EC_50_) and after stabilization of responses ((I_1_ + I_2_) EC_50_). The deactivation time constant (τ_off_) was measured in naïve receptors as decay of current after washout of 30–100 μM BzATP. The dose–response data were derived from 6–12 measurements per mutant per concentration and 33 to 194 measurements for the WT receptor per concentration. Ethidium bromide (EtBr) uptake (in % of control) was measured in cells expressing the WT and mutated receptors 100 s after stimulation with 100 µM BzATP; these data were derived from 3–7 experiments. Statistical significance (WT vs. mutants) was estimated by using one-way ANOVA followed by SNK or Dunnett’s test (* *p* < 0.05, ** *p* < 0.01); n.f., nonfunctional receptor; n.d., not determined.

## Abbreviations

ATP	Adenosine 5′-triphosphate
BzATP	2′,3′-O-(Benzoyl-4-benzoyl)-ATP
EC_50_	Half maximal effective concentration
HEK293T	Human embryonic kidney 293T
TM1	Transmembrane domain 1
TM2	Transmembrane domain 2
WT	Wild-type

## References

[1] Adinolfi E., Pizzirani C., Idzko M., Panther E., Norgauer J., Di Virgilio F., Ferrari D. (2005). P2X(7) receptor: Death or life?. Purinergic Signal..

[2] Burnstock G. (2006). Pathophysiology and therapeutic potential of purinergic signaling. Pharmacol. Rev..

[3] Surprenant A., North R.A. (2009). Signaling at purinergic P2X receptors. Annu. Rev. Physiol..

[4] Stojilkovic S.S. (2009). Purinergic regulation of hypothalamopituitary functions. Trends Endocrinol. Metab..

[5] Khakh B.S., North R.A. (2012). Neuromodulation by extracellular ATP and P2X receptors in the CNS. Neuron.

[6] Coddou C., Yan Z., Obsil T., Huidobro-Toro J.P., Stojilkovic S.S. (2011). Activation and regulation of purinergic P2X receptor channels. Pharmacol. Rev..

[7] Surprenant A., Rassendren F., Kawashima E., North R.A., Buell G. (1996). The cytolytic P2Z receptor for extracellular ATP identified as a P2X receptor (P2X7). Science.

[8] Rassendren F., Buell G.N., Virginio C., Collo G., North R.A., Surprenant A. (1997). The permeabilizing ATP receptor, P2X7. Cloning and expression of a human cDNA. J. Biol. Chem..

[9] Kopp R., Krautloher A., Ramirez-Fernandez A., Nicke A. (2019). P2X7 Interactions and Signaling—Making Head or Tail of It. Front. Mol. Neurosci..

[10] Di Virgilio F., Dal Ben D., Sarti A.C., Giuliani A.L., Falzoni S. (2017). The P2X7 Receptor in Infection and Inflammation. Immunity.

[11] Vavra V., Bhattacharya A., Zemkova H. (2011). Facilitation of glutamate and GABA release by P2X receptor activation in supraoptic neurons from freshly isolated rat brain slices. Neuroscience.

[12] Illes P., Khan T.M., Rubini P. (2017). Neuronal P2X7 Receptors Revisited: Do They Really Exist?. J. Neurosci..

[13] Kaczmarek-Hajek K., Zhang J., Kopp R., Grosche A., Rissiek B., Saul A., Bruzzone S., Engel T., Jooss T., Krautloher A. (2018). Re-evaluation of neuronal P2X7 expression using novel mouse models and a P2X7-specific nanobody. eLife.

[14] Burnstock G. (2016). P2X ion channel receptors and inflammation. Purinergic Signal..

[15] Klaft Z.J., Schulz S.B., Maslarova A., Gabriel S., Heinemann U., Gerevich Z. (2012). Extracellular ATP differentially affects epileptiform activity via purinergic P2X7 and adenosine A1 receptors in naive and chronic epileptic rats. Epilepsia.

[16] Nurkhametova D., Kudryavtsev I., Guselnikova V., Serebryakova M., Giniatullina R.R., Wojciechowski S., Tore F., Rizvanov A., Koistinaho J., Malm T. (2019). Activation of P2X7 Receptors in Peritoneal and Meningeal Mast Cells Detected by Uptake of Organic Dyes: Possible Purinergic Triggers of Neuroinflammation in Meninges. Front. Cell. Neurosci..

[17] Sperlagh B., Illes P. (2014). P2X7 receptor: An emerging target in central nervous system diseases. Trends Pharmacol. Sci..

[18] Stokes L., Spencer S.J., Jenkins T.A. (2015). Understanding the role of P2X7 in affective disorders-are glial cells the major players?. Front. Cell. Neurosci..

[19] Garre J.M., Silva H.M., Lafaille J.J., Yang G. (2020). P2X7 receptor inhibition ameliorates dendritic spine pathology and social behavioral deficits in Rett syndrome mice. Nat. Commun..

[20] Koldej R.M., Perera T., Collins J., Ritchie D.S. (2020). Association between P2X7 Polymorphisms and Post-Transplant Outcomes in Allogeneic Haematopoietic Stem Cell Transplantation. Int. J. Mol. Sci..

[21] Wang X., Arcuino G., Takano T., Lin J., Peng W.G., Wan P., Li P., Xu Q., Liu Q.S., Goldman S.A. (2004). P2X7 receptor inhibition improves recovery after spinal cord injury. Nat. Med..

[22] Cekic C., Linden J. (2016). Purinergic regulation of the immune system. Nat. Rev. Immunol..

[23] Danquah W., Meyer-Schwesinger C., Rissiek B., Pinto C., Serracant-Prat A., Amadi M., Iacenda D., Knop J.H., Hammel A., Bergmann P. (2016). Nanobodies that block gating of the P2X7 ion channel ameliorate inflammation. Sci. Transl. Med..

[24] Choi G.B., Yim Y.S., Wong H., Kim S., Kim H., Kim S.V., Hoeffer C.A., Littman D.R., Huh J.R. (2016). The maternal interleukin-17a pathway in mice promotes autism-like phenotypes in offspring. Science.

[25] Horvath G., Otrokocsi L., Beko K., Baranyi M., Kittel A., Fritz-Ruenes P.A., Sperlagh B. (2019). P2X7 Receptors Drive Poly(I:C) Induced Autism-like Behavior in Mice. J. Neurosci..

[26] Nicke A., Baumert H.G., Rettinger J., Eichele A., Lambrecht G., Mutschler E., Schmalzing G. (1998). P2X1 and P2X3 receptors form stable trimers: A novel structural motif of ligand-gated ion channels. EMBO J..

[27] North R.A. (2002). Molecular physiology of P2X receptors. Physiol. Rev..

[28] Kawate T., Michel J.C., Birdsong W.T., Gouaux E. (2009). Crystal structure of the ATP-gated P2X(4) ion channel in the closed state. Nature.

[29] Browne L.E., Compan V., Bragg L., North R.A. (2013). P2X7 receptor channels allow direct permeation of nanometer-sized dyes. J. Neurosci..

[30] Harkat M., Peverini L., Cerdan A.H., Dunning K., Beudez J., Martz A., Calimet N., Specht A., Cecchini M., Chataigneau T. (2017). On the permeation of large organic cations through the pore of ATP-gated P2X receptors. Proc. Natl. Acad. Sci. USA.

[31] McCarthy A.E., Yoshioka C., Mansoor S.E. (2019). Full-Length P2X7 Structures Reveal How Palmitoylation Prevents Channel Desensitization. Cell.

[32] Pippel A., Stolz M., Woltersdorf R., Kless A., Schmalzing G., Markwardt F. (2017). Localization of the gate and selectivity filter of the full-length P2X7 receptor. Proc. Natl. Acad. Sci. USA.

[33] Karasawa A., Kawate T. (2016). Structural basis for subtype-specific inhibition of the P2X7 receptor. eLife.

[34] Kawate T., Robertson J.L., Li M., Silberberg S.D., Swartz K.J. (2011). Ion access pathway to the transmembrane pore in P2X receptor channels. J. Gen. Physiol..

[35] Egan T.M., Khakh B.S. (2004). Contribution of calcium ions to P2X channel responses. J. Neurosci..

[36] Samways D.S., Khakh B.S., Egan T.M. (2012). Allosteric Modulation of Ca2+ flux in Ligand-gated Cation Channel (P2X4) by Actions on Lateral Portals. J. Biol. Chem..

[37] Samways D.S., Khakh B.S., Dutertre S., Egan T.M. (2011). Preferential use of unobstructed lateral portals as the access route to the pore of human ATP-gated ion channels (P2X receptors). Proc. Natl. Acad. Sci. USA.

[38] Samways D.S., Egan T.M. (2007). Acidic amino acids impart enhanced Ca^2+^ permeability and flux in two members of the ATP-gated P2X receptor family. J. Gen. Physiol..

[39] Yan Z., Liang Z., Obsil T., Stojilkovic S.S. (2006). Participation of the Lys313-Ile333 sequence of the purinergic P2X4 receptor in agonist binding and transduction of signals to the channel gate. J. Biol. Chem..

[40] Rokic M.B., Stojilkovic S.S., Vavra V., Kuzyk P., Tvrdonova V., Zemkova H. (2013). Multiple roles of the extracellular vestibule amino acid residues in the function of the rat P2X4 receptor. PLoS ONE..

[41] Virginio C., MacKenzie A., North R.A., Surprenant A. (1999). Kinetics of cell lysis, dye uptake and permeability changes in cells expressing the rat P2X7 receptor. J. Physiol..

[42] Roger S., Pelegrin P., Surprenant A. (2008). Facilitation of P2X7 receptor currents and membrane blebbing via constitutive and dynamic calmodulin binding. J. Neurosci..

[43] Roger S., Gillet L., Baroja-Mazo A., Surprenant A., Pelegrin P. (2010). C-terminal calmodulin-binding motif differentially controls human and rat P2X7 receptor current facilitation. J. Biol. Chem..

[44] Khadra A., Tomic M., Yan Z., Zemkova H., Sherman A., Stojilkovic S.S. (2013). Dual Gating Mechanism and Function of P2X7 Receptor Channels. Biophys. J..

[45] Allsopp R.C., Evans R.J. (2015). Contribution of the Juxtatransmembrane Intracellular Regions to the Time Course and Permeation of ATP-gated P2X7 Receptor Ion Channels. J. Biol. Chem..

[46] Yan Z., Li S., Liang Z., Tomic M., Stojilkovic S.S. (2008). The P2X7 receptor channel pore dilates under physiological ion conditions. J. Gen. Physiol..

[47] Jiang L.H., Rassendren F., Spelta V., Surprenant A., North R.A. (2001). Amino acid residues involved in gating identified in the first membrane-spanning domain of the rat P2X(2) receptor. J. Biol. Chem..

[48] Roberts J.A., Evans R.J. (2006). Contribution of conserved polar glutamine, asparagine and threonine residues and glycosylation to agonist action at human P2X1 receptors for ATP. J. Neurochem..

[49] Allsopp R.C., El Ajouz S., Schmid R., Evans R.J. (2011). Cysteine scanning mutagenesis (residues Glu52-Gly96) of the human P2X1 receptor for ATP: Mapping agonist binding and channel gating. J. Biol. Chem..

[50] Du J., Dong H., Zhou H.X. (2012). Gating mechanism of a P2X4 receptor developed from normal mode analysis and molecular dynamics simulations. Proc. Natl. Acad. Sci. USA.

[51] Digby H.R., Roberts J.A., Sutcliffe M.J., Evans R.J. (2005). Contribution of conserved glycine residues to ATP action at human P2X1 receptors: Mutagenesis indicates that the glycine at position 250 is important for channel function. J. Neurochem..

[52] Roberts J.A., Evans R.J. (2007). Cysteine substitution mutants give structural insight and identify ATP binding and activation sites at P2X receptors. J. Neurosci..

[53] Rassendren F., Buell G., Newbolt A., North R.A., Surprenant A. (1997). Identification of amino acid residues contributing to the pore of a P2X receptor. EMBO J..

[54] Boue-Grabot E., Emerit M.B., Toulme E., Seguela P., Garret M. (2004). Cross-talk and co-trafficking between rho1/GABA receptors and ATP-gated channels. J. Biol. Chem..

[55] Boue-Grabot E., Barajas-Lopez C., Chakfe Y., Blais D., Belanger D., Emerit M.B., Seguela P. (2003). Intracellular cross talk and physical interaction between two classes of neurotransmitter-gated channels. J. Neurosci..

[56] Lukacs G.L., Segal G., Kartner N., Grinstein S., Zhang F. (1997). Constitutive internalization of cystic fibrosis transmembrane conductance regulator occurs via clathrin-dependent endocytosis and is regulated by protein phosphorylation. Biochem. J..

[57] Caohuy H., Jozwik C., Pollard H.B. (2009). Rescue of DeltaF508-CFTR by the SGK1/Nedd4-2 signaling pathway. J. Biol. Chem..

